# Scheme of Effective Epidemiological Investigations in *Trichinella* Outbreaks on Pig Farms

**DOI:** 10.3390/foods12061320

**Published:** 2023-03-20

**Authors:** Ewa Bilska-Zając, Weronika Korpysa-Dzirba, Aneta Bełcik, Jacek Karamon, Jacek Sroka, Tomasz Cencek

**Affiliations:** Department of Parasitology and Invasive Diseases, National Veterinary Research Institute, Partyzantow Avenue 57, 24-100 Pulawy, Poland

**Keywords:** *Trichinella* spp., epidemiological investigation, outbreak, pig farm

## Abstract

Trichinellosis is a parasitic, zoonotic disease caused by larvae of the genus *Trichinella*. Infection occurs via the consumption of raw or undercooked meat containing this parasite. Symptoms of the disease manifest as intestinal disorders, followed by facial swelling, fever, muscle pain and other symptoms, eventually leading to neurological and cardiac complications and even death. In Europe, trichinellosis is most often associated with the consumption of meat from wild boars, pigs and horses. In recent years, wild boars that are hunted illegally and not tested for *Trichinella* spp. have been the most common cause of trichinellosis in humans; however, there have also been cases where infected pigs have been the source of infection. When trichinellosis is suspected in humans, epidemiological measures are taken to identify the source. Similarly, an epidemiological investigation should be initiated whenever *Trichinella* spp. has been detected in pigs. However, commonly used actions do not provide sufficient data to determine the source of infection for pigs and to prevent further transmission. Therefore, in this article, we propose a scheme for effective epidemiological investigations into *Trichinella* outbreaks on pig farms that can help trace the transmission mechanisms of the parasite and that takes into account currently available testing tools. The proposed pathway can be easily adopted for epidemiological investigations in routine veterinary inspection work.

## 1. Introduction

Trichinellosis is a parasitic disease, of which the etiological factors are nematodes of the genus *Trichinella* spp. [1]. It is usually a severe zoonosis, meaning humans can become infected by eating meat containing the live larvae of parasites. In Europe, the disease most often occurs through the consumption of wild boar (*Sus scrofa*), pig (*Sus domestica*) and horse (*Equus caballus*) meat products and less often through meat products derived from other species [2]. *Trichinella* parasites spread between animals through the ingestion of meat tissue from animals infected by these nematodes. Vertical transmission of the parasite from mother to offspring is also possible [3]. These parasites can infect carnivores and omnivores but also herbivores. They have been found in mammals, birds and reptiles all over the world. *Trichinella* has so far been confirmed in about 150 animal species [4,5]. Thirteen *Trichinella* genotypes have been recognized to date, of which nine species have been identified. Based on molecular studies, four of them have been confirmed in Europe, namely *Trichinella spiralis*, *T. britovi, T. pseudospiralis* and *T. nativa* [6], among which the most common species are *T. spiralis* and *T. britovi.* All genotypes can cause trichinellosis, but the most common infections in Europe are related to *T. spiralis* [7,8].

In recent years, the majority of cases of human trichinellosis have been caused by the consumption of the infected meat of wild boars, which are hunted illegally and not examined for the presence of *Trichinella* spp. [9,10,11]. The increasing percentage of infected wild boars poses further risks to humans. The noted year by year cases of *Trichinella* infections in pigs population in some EU countries indicate that the problem exists [2]. The domestic and sylvatic cycles often overlap with common vectors, such as rodents, living in the fields in the summer and gathering on farms in the winter where they have access to food [12]. The vectors may have great importance in the transmission of *Trichinella* between farms and sylvatic environments [13]. The transmission caused by parasites may be exacerbated by farm owners who support the circulation of parasites through illegal actions (e.g., feeding pigs with waste from hunted animals or scraps from slaughter). Epidemiological investigations by veterinary personnel into the trichinellosis outbreaks in pig farms are important to prevent the further transmission of parasites and prevent human infection [14]. Such investigations are of particular importance in endemic areas and areas with a high concentration of pig production. Epidemiological investigations should provide answers to the most important question in an outbreak, i.e., what is the source of infection? To provide answers, certain actions must be undertaken. First, an appropriate epidemiological interview followed by confirmation of suspected sources should be carried out. In this article, we suggest carrying out epidemiological investigations in cases of *Trichinella* outbreak in pigs, while taking into account available testing tools.

### 1.1. Trichinellosis Infections in Europe

Trichinellosis is included in a list of zoonoses and zoonotic agents that are under mandatory annual monitoring according to Directive 2003/99/EC List A [15]. As reported by the European Food Safety Authority (EFSA), cases of human trichinellosis in Europe indicate that there is a continued threat of this parasitic zoonosis in some countries. Most of the cases recorded in previous decades were in Romania, Bulgaria, Latvia, Lithuania and Poland [16]. However, data from the last five years present a slightly different epidemiological situation, indicating a decreasing trend of human trichinellosis cases in Romania [16,17,18,19,20,21,22,23] (Table 1). In 2021, 77 confirmed cases of human trichinellosis were reported in 26 of the EU/EEA (European Union/European Economic Area) countries. Bulgaria and Croatia had the highest notification rates in the EU (0.42 cases per 100,000 in both countries), followed by Latvia (0.37 cases per 100,000) and Austria (0.11 cases per 100,000). Together, these four countries (Austria, Bulgaria, Croatia and Latvia) accounted for 80% of all confirmed trichinellosis cases reported at the EU level in 2021 [24]. Out of these 77 cases of human trichinellosis, 29 were acquired in Europe, 2 cases were acquired outside Europe, and 46 cases did not have a confirmed status of origin [24]. When a person becomes infected with *Trichinella* by consuming cured, untested meats and *Trichinella* spp. larvae are found in the residue of these products, the source of infection is easy to determine. However, the situation is not always this simple to solve. The biggest problem occurs when people become infected when traveling and there is no possibility of tracing these cases back to a possible source of infection. While food traceability is provided by the TRAde Control and Expert System (TRACES) for travel within EU countries, it is not always possible for countries outside of the EU. The occurrence of such a large number of cases with an unknown source demonstrates the considerable difficulties that can be encountered during epidemiological investigations [25].

### 1.2. Trichinella in Pigs in EU

According to the Commission Implementing Regulation (EU) 2015/1375 [14], all *Trichinella*-susceptible animals intended for human consumption in the EU, i.e., domestic pigs, wild boars and solipeds, should be tested for the presence of *Trichinella* larvae in the muscles unless carcasses have undergone a freezing treatment. The recommended method is the magnetic stirrer method for pooled sample digestion for *Trichinella* spp., as described in the ISO 18743/2015 standard [26]. However, an equivalent method to the recommended one may be used (Commission Implementing Regulation (EU) 2015/1375). In 2021, of the more than 246 million pigs reared in European countries, over 216 million were tested for the presence of *Trichinella* in their carcasses [27]. Of that number, 120 pigs tested positive for this parasite; therefore, a prevalence of 0.00005% and rate of about 0.49 per million reared pigs was detected [27]. The prevalence of *Trichinella* spp. in the EU seems to show a downward trend (Table 2). Importantly, all of the positive pigs originated from farms without controlled housing conditions (NRCHC), and no positive cases were observed on farms with controlled housing conditions (RCHC) [28]. Within the EU, the infected pigs were from Bulgaria, Croatia, Poland, Romania and Spain; sporadic infections are documented in other countries [29]. EFSA identified that a major risk factor for *Trichinella* infections is not raising domestic pigs under controlled housing conditions. Backyard or free-range pigs are the biggest risk for *Trichinella* infection. Breeding conditions are important as they affect the number of pig infections that occur, and this is directly related to the risk of trichinellosis infecting humans. Therefore, suitable control of herds by the Veterinary Inspection is important. In EU countries, such control is carried out in accordance with the Veterinary Inspection Service and is mandatory [30,31]. Unfortunately, in many countries around the world, requirements for breeding or animal welfare are very sparse, meaning that veterinary control is negligible or easily avoided [29]. As mentioned, compliance with breeding requirements, proper control of farms and owners’ awareness of possible routes of *Trichinella* parasite infection in pigs constitute an important barrier to prevent the transmission of these parasites. This is supported by the fact that there are no reports of *Trichinella* infection on farms with pigs raised in controlled housing conditions [32]. For example, in Bulgaria or Croatia, there has been an increasing number of RCHC pigs and an increased control of the slaughter of NRCHC pigs during the last few years. These measures, in combination with trichinellosis awareness and farmers-education activities, may have contributed to a reduction in the parasite biomass in domestic habitats and a reduction in the probability of acquiring an infection for humans [29]. According to EFSA, the identification of *Trichinella* larvae at the species level in 2021 confirmed that *T. spiralis* was most prevalent in pigs (83% of positive cases), and *T. britovi* was reported in 13% [27]. Similar data were published based on data collected from EU countries in 2009 by EURLP [7]. *T. pseudospiralis* was reported in only one out of sixty pigs (1.8%), for which the *Trichinella* species was available; these data confirmed the low prevalence of this species in the examined animals [33].

### 1.3. Epidemiologic Epidemiological Investigation on Farms with Trichinella Infected Pigs

When a *Trichinella* infection is found in pigs, it is particularly important to identify the source of the infection for the animals and stop further transmission of the parasite to other pigs and the surrounding areas. For this, an epidemiological investigation is used, which is a complex procedure that includes only a few stages [14].

#### 1.3.1. Epidemiological Interview

The first stage of an epidemiological investigation is an epidemiological interview, which if properly conducted, can lead to the identification of the source of the parasite. First, it is necessary to carefully analyze the number of animals raised in a given herd. The collection of data on the herd size, husbandry conditions, animal welfare and the number of sows and fattening pigs in the herd can facilitate an epidemiological investigation and indirectly indicate the routes by which *Trichinella* can infect pigs. It is necessary to analyze all potential routes of *Trichinella* infection in pigs [34]. Therefore, particular attention is paid to pigs’ diet, the type of feed and its origin. Herd owners aware of *Trichinella* spp. transmission routes most often do not engage in the illegal activity of introducing feed of unknown origin. On such farms, trichinellosis occurs rather incidentally and infections most often can be caused through vectors such as rodents [35,36]. Other sources of trichinellosis will be suspected when herd owners engage in illegal activities, such as feeding pigs with slaughter waste, waste after shooting wild boars or other wild animals, or carcasses from fur animal farms [37,38]. Therefore, during an epidemiological interview, it is highly important to review the surrounding areas and to check whether there are fur farms, garbage dumps (presence of rodents) in the vicinity, or if there are hunting grounds in the neighborhood [36,37]. As mentioned, during epidemiological investigations carried out on pig farms, the assessment of the conditions in livestock buildings is also important, especially in terms of access to rodents, which can act as a vector for these parasites. There is a high risk of *Trichinella* infection in pigs on organic farms or farms with outdoor access, which is related to the animals’ contact to the outside environment and, thus, to *Trichinella* vectors [35,39]. In most farms where *Trichinella*-infected pigs are found, the presence of rats can be observed. From the data presented on the prevalence of trichinellosis in rats (*Rattus*) in Poland, it is clear that these rodents can have a large impact on the persistence of trichinellosis infection on a pig farm. In addition, they can be an important vector transmitting the parasite to surrounding farms or to the environment [39]. Therefore, for diagnostic purposes, rodent trapping campaigns should be carried out on farms. The collected rodents’ carcasses should be tested according to the digestion method for *Trichinella* presence [26]. The *Trichinella* larvae discovered in rodents should be tested for species identification [40]. Such data can be valuable when *Trichinella* transmission by vectors is suspected (rodents) [35]. Collecting all possible data during the epidemiological interview may facilitate the identification of the source of infection for pigs. In many cases, the cause of a *Trichinella* infection on a given farm may already be known at this stage of the epidemiological interview. However, in other situations, additional steps such as the serological testing [41,42,43] of suspected animals, confirmatory testing by muscle tissue digestion, and genetic testing of detected *Trichinella* larvae to determine their species are carried out [44].

#### 1.3.2. Serological Investigations

Serological tests reveal the epidemiological background and indicate the possible presence of an infection in the herd. Most often, rapid diagnostic tests that detect the presence of antibodies against *Trichinella* spp. are used to analyze trichinellosis outbreaks [41]. Such methods include the ELISA test, which is useful primarily for screening and Western Blot and is also used as a reference test [45]. The commonly used commercial ELISA tests can help to identify IgG antibodies against excretory–secretory (E-S) antigens of *Trichinella* spp. muscle larvae (ML) [46]. It should be noted that *T. spiralis* larvae settle in pig’ muscle tissue at around day 17 after infection [47]. In contrast, the appearance of IgG antibodies in infected pigs occurs 3 weeks after *T. spiralis* infection at the earliest [48]. The time required to develop a specific IgG antibody response in *Trichinella*-infected pigs is correlated with both the dose of infection and the intensity of *Trichinella* infection in muscles [49,50]. Many studies indicate even a later appearance of IgG in the blood (30 days after invasion by *T. spiralis*). In the case of *T. britovi* or *T. pseudospiralis* infection in pigs, seroconversion is picked up even later than in the case of *T. spiralis* infections, as late as 36 days after infection [51,52]. This causes difficulties in detecting *Trichinella* invasions at earlier stages. In this period, for invasions at 30 (*T. spiralis*)/36 (*T. britovi*) days after invasion, the ELISA results can be a false negative [42,43,46,48,53,54,55,56,57,58]. This is especially important because once the larvae are in the muscle tissue, they become invasive to the next host (including humans). In epidemiological investigations of *Trichinella* outbreaks on pig farms, the sera should be collected from each pig and then the ELISA test should be performed. However, the researchers should remember that when early trichinellosis is suspected or uncertain results are obtained with the ELISA test, sera should be taken from pigs a second time after around 30 days, and the ELISA test should be performed again. Drawing on our own experience of investigations carried out in *Trichinella* outbreaks, it appears that commercial ELISA tests do not show sufficient sensitivity to confidently infer the existence of *Trichinella* spp. infection in the animals under investigation. Therefore, optionally, a much more sensitive Western Blot (WB) test can be performed to confirm the ELISA test results [41].

#### 1.3.3. Removing Other Infected Pigs from Farms

Positive serological tests conducted in trichinellosis outbreaks provide the basis for the next steps, i.e., to isolate infected animals and eliminate them from the herd. The sanitary slaughter of infected animals should be carried out at a designated slaughterhouse. The final verification of serological results is the post-slaughter examination of a sample of muscle tissue taken from predilection sites using the reference magnetic stirrer digestion method (ISO 18043) [26]. The detection of *Trichinella* larvae results in the carcass being declared “unfit for consumption” and sent for disposal (as a I category waste) [30]. Parasite larvae isolated by digestion are subjected to genetic testing to determine the species [44,59]. However, owners sometimes send animals to distant slaughterhouses to avoid losses in hopes of receiving a negative test result for *Trichinella* spp. infection. This further complicates investigations into trichinellosis outbreaks.

#### 1.3.4. Species Identification of Detected Larvae

The identification of the species of *Trichinella* larvae is determined using molecular methods, primarily a polymerase chain reaction (PCR) and its modifications [44]. The most common, yet fast and reliable is the multiplex PCR method, recommended by the European Union Reference Laboratory for Parasites (EURLP) [60]. This method is based on the amplification of selected fragments of genes (ESV, ITS1, ITS2), followed by species recognition [44,61]. First, the DNA from collected single larva is extracted using commercial kits (Promega Corporation, Madison, WI, USA// Qiagen Corporation, Hilden, Germany), and then the DNA is used for the amplification of selected fragments of genes. Species recognition is based on the size of the obtained amplification products visualized during the horizontal electrophoresis process under UV light. However, this step may also be conducted by using other molecular methods available in the literature to achieve species recognition, including Transcription–PCR (RT–PCR), Restriction Fragment Length Polymorphism–PCR (RFLP–PCR), Random Amplification of Polymorphic DNA (RAPD–PCR), Single Strand Conformation Polymorphism (SSCP–PCR), and sequencing of the chosen DNA fragments [40,62,63,64,65]. Recently, matrix-assisted laser desorption ionization time-of-flight mass spectrometry (MALDI–TOF–MS) was also applied to identify species of *Trichinella* nematodes; good resolution, fast lab work and low-cost testing were achieved [66]. At this stage of the epidemiological investigation, species identification can sometimes lead to the exclusion of the suspected source of trichinellosis. It may happen in the case of the occurrence of two different species in an infected pig and in a suspected source of infection, e.g., vector. For example, at one pig farm in Estonia, four *Trichinella* spp. infected pigs were detected and it was suspected that the source of trichinellosis for these pigs were rats living on the farm. Species identification indicated, however, that the rats were infected with *T. britovi*, while *T. spiralis* was found in the pigs [67,68]. Generally, the most common scenario is the detection of the same *Trichinella* species both in the infected pig and in the suspected source (vectors, leftovers from hunting, carcasses from fur farms). As mentioned above, the most commonly detected species in pigs in Europe is *T. spiralis*; however, outbreaks caused by *T. britovi* and *T. pseudospiralis* infections in pigs have also been reported [69,70,71,72,73]. The species with which a pig is infected is determined by the source from which these animals can become infected (leftovers from hunting, feeding on farm slaughter waste, etc.). Therefore, genetic studies leading to the recognition of larvae species are also important from an epidemiological point of view. In general, it is difficult to analyze the occurrence of this parasite in other hosts during an ongoing outbreak of trichinellosis. Therefore, it is important to collect epidemiological data on the occurrence of individual *Trichinella* species in different animals (also sylvatic) in a given area (country) when performing, for example, annual monitoring. The collecting of such data provides a good background for performing an analysis, identifying routes of transmission and determining the source of infection for animals in a given herd.

However, most often, for the purposes of epidemiological investigations into outbreaks, mere determination of the species of *Trichinella* causing the infection is insufficient. Most often, the species found in infected pigs are identical to those found in potential vectors. Modern molecular techniques based on the analysis of selected gene fragments can be helpful in such situations, allowing the population to be accurately characterized and subpopulations to be distinguished.

#### 1.3.5. Differentiation of Isolates of the Same Species Using Available Molecular Epidemiology Tools

This stage is the most difficult and is rarely technically performed; however, it is the most effective method that can be used to confirm or rule out the source of trichinellosis. In cases of *Trichinella* spp. outbreaks, there is no standard method applicable to epidemiological investigations. However, scientific studies indicate that it is possible to distinguish between larva isolates of a single species. Potentially helpful methods include analyzing of microsatellite markers [74,75,76] and double digest restriction-site-associated DNA (ddRADseq) [77,78]. Microsatellite markers are randomly occurring, short repeated nucleotide sequences in the genome, most often located on autosomes [79,80]. Because they are characterized by a large variation in the number of repeats of a given microsatellite related to mutation frequency, they are a good indicator for detecting differences between closely related lineages [81,82]. The evolutionarily older a group is, the more frequent the mutations. Microsatellite DNA analyses are used to determine variations between populations as well as genetic variation within the populations under study. This method was successfully applied in an epidemiological investigation applied to an infection by *T. britovi* species [75]. *T. spiralis*, however, is a much more genetically homogeneous species and contains much less genetic variation [76]. This leads to difficulty in identifying genetic differences between isolates of *T. spiralis* larvae. However, distinguishing the isolates of *T. spiralis* is also possible using microsatellite analysis [74]. This procedure is time-consuming, however, and researchers must collect larvae from animals in the outbreak from vectors suspected of transmitting the parasites or other suspected sources when conducting an epidemiological investigation (e.g., feed). Next, the researcher must select at least 36 larvae per each isolate (sample) in the best condition, preferably live larvae, from which the whole DNA is extracted. Then, in this procedure, 7 microsatellite markers are amplified for 36 individual larvae from a single isolate. Each microsatellite marker is amplified in a separate PCR reaction [78]. Based on the analysis of the microsatellite markers, the genetic structure of each tested isolate is characterized and compared. As a result, based on the different genetic structures of the tested isolates, one is able to distinguish them from each other.

The differentiation of isolates of the same species can also be achieved using a recently developed tool called the Trich-tracker method. This method is based on ddRADseq and bioinformatics analysis, which appears to be far less time- and labor-intensive and cost-effective. The methodology is based on the ddRADseq technique, during which DNA libraries are created using restriction enzymes (they cut specific motifs wherever they occur in the genome) [79,83]. Such DNA libraries contain the sequences of multiple random loci distributed throughout the genome under analysis. Simultaneous sequencing of all such fragments provides information on thousands of loci (which includes a large amount of genetic data) but not on the entire genome (which reduces the cost of the study) [78,81]. The raw genetic data obtained during sequencing can be studied for single nucleotide polymorphisms (SNPs), which are valuable for understanding organism epidemiology, evolutionary history and ecology [79]. This technique provides excellent resolution when determining population genetic structure and phylogeographic history [83]. To use this procedure in epidemiological investigations, researchers must collect larvae from animals in the outbreak from vectors suspected of transmission of the parasites or other suspected sources (e.g., feed). This is similar in the case of microsatellites analysis. However, larvae from the given isolate are analyzed as a pool using Trich-tracker, which decreases the time required for lab work. The DNA is extracted separately from the prepared isolates (pooled larvae from one animal), and a library for new generation sequencing (NGS) is prepared. Then, the obtained NGS raw sequence data are used for phylogenetic analysis or genetic structure analysis; both of the analyses may be used to distinguish isolates from one another. This procedure is characterized by less labor in the laboratory, meaning results can be obtained within one week [83].

The use of both the proposed methods make it possible to exclude or confirm the suspected source of trichinellosis, thus ensuring its removal and preventing further spread of the parasite. These techniques may also be useful in situations where a given meat product containing *Trichinella* larvae is suspected to be a source of infection in humans. Similarly, they can be used to track where the product originated from in case of unknown origin. Therefore, it is highly important to collect data on the occurrence of particular *Trichinella* species in the study area, as mentioned earlier. The best way to do this is to create a bank of isolates that can be used for the collection of genetic data in future epidemiological studies.

**Figure 1:**
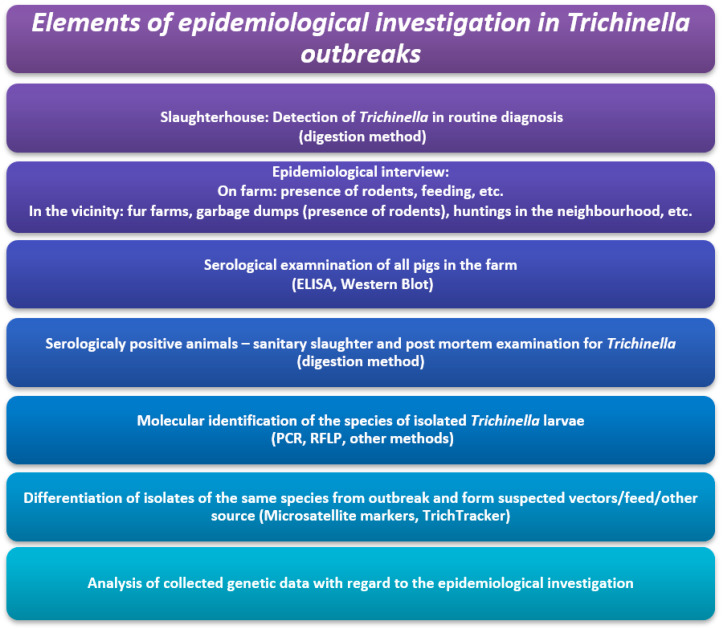
Elements of epidemiological investigation in *Trichinella* outbreaks.

The proposed steps of the epidemiological investigation for *Trichinella* outbreaks are presented in Figure 1.

## 2. Conclusions

As indicated by the number of cases detected over the last few years, trichinellosis is still a significant problem in the context of food safety; therefore, increased attention should be paid to the circulating of this parasite in natural and domestic environments. All human cases of this zoonosis reported inside and outside of the EU in recent years confirm the danger of eating meat that is of unknown origin or insufficiently heat treated. Finding the source of infection is important, especially as the cause is unknown for most of the recorded positive cases. The scheme proposed in this article for the investigation of trichinellosis in pig meat or meat products could be used routinely in epidemiological investigations. This would provide insight into the mechanism of trichinellosis transmission on farms and between animals in the natural environment. Thus, it would help remove the sources of trichinellosis and prevent human infections.

## Figures and Tables

**Table 1:** Human trichinellosis in EU/EEA between 2017 and 2021 (adapted from EFSA (European Food Safety Authority); ECDC (European Centre for Disease Prevention and Control). The European Union One Health 2020 Zoonoses. *EFSA J.* **2021**, *19*, 6971) [23].

Year	2017	2018	2019	2020	2021
Total number of confirmed cases	168	66	97	117	77
Total number of confirmed cases/100,000 population (notification rates)	0.03	0.01	0.02	0.03	0.02
Number of reporting countries	27	27	27	26	26
Infections acquired in the EU	81	18	26	99	29
Infections acquired outside the EU	2	1	2	2	2
Unknown travel status or unknown country of infection	85	47	69	16	46
Number of outbreak-related cases	199	114	44	119	2
Total number of outbreaks	11	10	5	6	1

**Table 2:** *Trichinella* spp. infections in pigs in EU, as reported by EFSA, between 2017 and 2021.

Year	2017	2018	2019	2020	2021
Domestic pigs raised under control housing condition					
Number of tested samples	55,177,802	55,989,292	73,633,900	77,794,786	72,227,074
% of positive samples (N)	0 (0)	0 (0)	0 (0)	0 (0)	0 (0)
Domestic pigs not raised under control housing condition					
Number of tested samples	124,689,434	152,922,322	145,213,445	139,637,631	161,129,635
% of positive samples (N)	0.0002 (224)	0.0003 (384)	0.00015 (219)	0.0001 (179)	0.0001 (120)

## Data Availability

Data is contained within the article.
